# Rural Adults’ Perspectives on School Food in a North Carolina County

**DOI:** 10.5888/pcd12.140484

**Published:** 2015-04-23

**Authors:** Jayne K. Jeffries, Linden M. Thayer, Heidi Hennink-Kaminski, Seth M. Noar

**Affiliations:** Author Affiliations: Linden M. Thayer, The Gillings School of Global Public Health, University of North Carolina at Chapel Hill, Chapel Hill, North Carolina; Heidi Hennink-Kaminski, Seth M. Noar, School of Journalism and Mass Communications, University of North Carolina at Chapel Hill, Chapel Hill, North Carolina.

## Abstract

**Introduction:**

To address alarming rates of youth obesity, multiple stakeholder perspectives must be understood and considered when developing nutrition interventions. The purpose of this qualitative study was to examine adults’ perceptions of school food in rural North Carolina and their opinions about potential changes to encourage students to eat more fruits and vegetables in school meals.

**Methods:**

We conducted semistructured key informant interviews by telephone from February through March 2013 to determine adult opinions regarding elementary school food and child health. Participants included parents, teachers, school administrators, and a cafeteria staff member. Interview transcripts were thematically analyzed using Dedoose qualitative analysis software.

**Results:**

Four themes emerged from key informant interviews regarding school meals and increasing fruit and vegetable consumption: 1) schools are an appropriate place for nutritious food, 2) current school food is bland and unappealing, 3) school cafeterias can use simple strategies to increase fruit and vegetable intake, and 4) federal school meal guidelines are perceived as barriers to increased fruit and vegetable intake during school meals.

**Conclusion:**

Study findings suggest that training and support for cafeteria staff on healthy food preparation and presentation are critical and that there should be a “meeting in the middle” between child appeal and health. Nutritious *and* appealing school food options may have the potential to greatly increase fruit and vegetable consumption in rural elementary schools in North Carolina.

## Introduction

Obesity contributes to leading causes of death in the United States, including heart disease, stroke, diabetes, and certain cancers (1). Eating healthy foods such as fresh fruits and vegetables can help prevent weight gain or maintain weight and reduce the risk of many chronic diseases (2,3). Studies demonstrate that rural children are more likely to consume diets high in unhealthy fats and sugar and low in fruits and vegetables (4) and are more likely to be obese than their urban counterparts (5). Recently, the odds of being obese in North Carolina were 50% higher among rural children than urban children, with nearly 14% of rural children exhibiting early risk factors for diabetes and heart disease (6). A lack of quality supermarkets and greater dispersion of food outlets could account for this disparity, meaning high-quality food products (eg, fresh fruits and vegetables) can be more expensive and more difficult for families in rural areas to obtain (7). One way to mitigate this potential access problem is to intervene with schools and adults, 2 key influences on a child’s eating behaviors.

Schools are recognized as ideal settings to address economic and social barriers to fruit and vegetable consumption because most children aged 6 to 18 years, regardless of demographic characteristics or geographic location, regularly attend school. Therefore, schools are in a unique position to influence and promote consumption of fruits and vegetables in this population (8). A study focused on educators and their motivations to increase fruit and vegetable consumption among students suggested that teachers perceive themselves to be parents at school and stress the importance of helping students cultivate healthy eating habits (9). Obesity prevention studies, such as 5-a-Day Power Plus (10), Food Dudes (11), Planet Health (12), and Child and Adolescent Trial for Cardiovascular Health (13), were conducted in school settings, but few published school-based diet interventions focused specifically on rural populations or were implemented after the Healthy, Hunger-Free Kids Act of 2010 (HHFKA).

HHFKA governs child nutrition programs and includes new nutrition standards for school meals, including the reduction of sodium and the percentage of calories from saturated fat and a significant increase in fiber (14). As of the 2013–2014 school year, all students receiving a reimbursable school lunch are required to take a minimum of 3 food components, one of which must be a fruit or vegetable (14). Before HHFKA, students were not required to take a fruit or a vegetable, and there were no limits on the sodium or calorie content of foods.

The effect the new standards will have on health outcomes for rural children is unclear. A recent study suggested the new standards increase student exposure to fruits and vegetables only in urban communities (15); little is known about rural communities’ reaction to, and perceptions of, school food post-HHFKA. Adult and student perceptions of ways to increase fruit and vegetable consumption among elementary school children before HHFKA included enhancing liking for vegetables, increasing availability of fruits and vegetables at home, and teaching how to prepare fruits and vegetables (16). Our study is unique because it examines adults’ perceptions of school food in rural North Carolina post-HHFKA, and its findings can support school-based changes.

The socio-ecological framework (SEF) highlights the need to understand determinants of child fruit and vegetable consumption on various social, physical, and political levels. The literature suggests dietary change interventions should address multiple levels of SEF to produce the greatest impact (17). SEF has 4 core levels (individual, interpersonal, community, and societal) (18), which have direct and interactive effects on health behaviors (17). A 2010 qualitative study demonstrated that parents, teachers, and students all identified adults, friends, and schools as influencers of children’s healthy behaviors (19). Therefore we must understand the perspective of stakeholder groups at different SEF levels that influence children’s obesity-related behaviors (20). To achieve this goal, we conducted key informant interviews with school personnel, parents, and cafeteria staff to inform the development of an intervention to increase fruit and vegetable consumption in rural school lunches because of adults’ perceived influence on children’s decision-making. The objectives of this research were 1) to assess stakeholder perceptions of elementary school food, and 2) to understand stakeholder beliefs about ways to increase children’s consumption of fruits and vegetables in school meals.

## Methods

### Participants

Four rural North Carolina elementary schools in Rockingham County were selected for a pilot program to increase fruit and vegetable consumption during school lunch. These schools all had a high proportion of students classified as overweight or obese, and most students’ families were classified as being of low socioeconomic status. Twenty-four adults were purposively recruited because of their roles in providing or supporting school lunch at these elementary schools. Participants included parents or guardians of children attending a pilot school, elementary school teachers, school administrators, and a school cafeteria staff person. School principals were recruited first and asked to recommend adults who might have particular insights into school food. The selected informants were also asked to recommend others that fit our inclusion criteria. We adopted this sampling strategy because we deemed it the most feasible way to reach geographically dispersed households. Of the 24 adults contacted, 17 (71.0%) agreed to participate. Seven adults (4 teachers and 3 school cafeteria staff members) declined because of time constraints. The final sample included 3 parents or guardians, 8 teachers, 5 school administrators, and 1 school cafeteria staff person. Fourteen (82.0%) of the participants were women. Years of experience as a teacher or administrator ranged from 7 to 33 years. The age of participants ranged from 28 to 70 years (mean, 43 y; standard deviation, 12 y).

### Procedure

The research team developed an interview guide focused on school food, child health, and school communication. To understand the multiple determinants of children’s consumption of fruits and vegetables, we used the SEF as the basis for the interview guide. Interview questions addressed different levels of the SEF. For example: “When I say ‘school food’ what comes to mind?” is an individual-level question, and “How can school lunch staff support efforts to raise a healthy child?” is an interpersonal- and community-level question. Questions such as “Do you have any ideas about how to encourage children to eat fruits and vegetables during school lunch?” spanned all levels of the SEF. The interview guide was pretested with 2 teachers and 3 parents unaffiliated with the pilot schools, and we made minor wording revisions to tailor questions to the audience.

All authors completed qualitative methods training and had at least 4 years of experience in qualitative research. This study was approved by the Institutional Review Board for human subjects at the University of North Carolina, Chapel Hill.

Two authors (J.K.J., L.M.T.) conducted semistructured key informant interviews from February through March 2013 via telephone. Interviews lasted approximately 45 minutes. The interviewer asked preselected questions and encouraged participants to raise relevant topics not included in the interview guide. Interviewers had no prior association with participants. All participants provided informed consent and agreed to audio recording of the interview. Participants did not receive monetary compensation for their time.

Interviewers performed data collection and discussed transcripts simultaneously to identify when no new themes were emerging in the interviews; recruitment concluded when new participants no longer revealed new concepts or information (21). Perspectives of different stakeholders were sought, but it quickly became clear that participants, regardless of their role (school personnel or parent), shared similar views. Three additional interviews were conducted to ensure no new information would be gained. Recruitment ceased after 17 interviews.

### Data analysis

Before analysis, all data were transcribed, deidentified, and checked for completeness and accuracy by a trained research assistant. The first author (J.K.J.) reviewed all transcripts while listening to the audio files before developing codes. Thematic analysis was considered most suitable for organizing the data because it moved analysis from a broad reading toward discovering patterns and developing themes (22). The data were primarily categorized and interpreted though coding. A codebook was developed based on interview guide themes (eg, school food, health concerns, increasing consumption of fruits and vegetables). During initial coding, memos were written about ideas for additional topical codes. These new topical codes were added to further explore themes in the data (eg, easier to eat, taste perception, visual perception, lack of choices). Codes were applied using Dedoose qualitative analysis software, version D. 4.7 (Dedoose).The same codebook was used for both aims of the study. To ensure the first author did not influence the interpretation of the data, all authors evaluated the codebook and memos. Any discrepancies were resolved by team consensus, and the codes were refined. After 2 rounds of coding, themes most relevant to the research objectives were reviewed and organized by SEF level, focusing on participant opinions about school food and suggestions to increase children’s consumption of fruits and vegetables. To reduce potential interpretation bias, the original transcripts were consulted to ensure findings were grounded in data. The themes presented in the Results section are derived from this thematic analysis of rural adult perceptions of school food. Verbatim quotes are presented to illustrate key participant perceptions.

## Results

### Stakeholder perceptions of elementary school food 

Two major themes emerged from participant descriptions of school food: 1) schools are an appropriate place for nutritious food, and 2) school food is bland and unappealing.

**Schools as an appropriate place for nutritious food.** Participants considered schools a natural place to promote wellness and provide nutritious meals to children. Participants believed schools are partly responsible for providing children with healthy foods. A school administrator emphasized students are at school 7 hours a day, and schools therefore have some responsibility for showing children how to live healthy lives, particularly through the types of foods offered at school.

Elementary schools provide breakfast and lunch to most low-income students Monday through Friday. Participants said that if those opportunities were not available, many students would be undernourished, because participants reported that many families in this county are unable to provide enough food at home. One cafeteria manager wished all food served in the cafeteria was free so no child would go hungry: “Nothing gives me more joy than to see the children come down the aisle, come down the line, and eat. Because there’s so many kids that doesn’t eat [at home] . . . So school lunches — I don’t know what would happen if we didn’t have them” (cafeteria staff manager).

Many participants believed the nutritional value of school food was adequate, especially for the cost. Teachers, in particular, said that school lunches were nutritious, but they also believed school nutrition services emphasized nutrition above taste and acceptability.

**Current school food is bland and unappealing.** Participants noted that recent federal policy changes negatively impacted school food’s taste and aesthetic appeal. Although some legislation was favorably regarded (particularly the required increases in availability of fruits and vegetables), other changes, such as the requirement to reduce sodium, were more unpopular because healthier foods are now considered bland and unappealing. “I mean, I think there is a point where they should offer the fruits and stuff, but give it some taste so it at least tastes decent. I don’t disagree with healthy foods, but I think there should be a meet in the middle kind of thing” (teacher).

The idea of taste “meeting in the middle” with nutritional value was common. Participants believed healthy food also should also be flavorful, even if it forfeited some health benefits. One teacher suggested adding cheese to broccoli as an example of “meeting in the middle.”

A cafeteria staff member expressed her understanding that federal school meal legislation is meant to better child health.

“You know, some of the food is a little bland because we don’t add anything to it. And that’s the way she [child nutrition director] wants, that’s the way they [legislators] want it to be. ‘Cause there’s obesity, you know? And that’s okay, some things are good. I’m learning to eat some things without salt” (cafeteria staff manager).

Preparing foods “the way . . . they want it to be” acknowledges changes in food preparation methods necessitated by the HHFKA. The cafeteria staff manager interviewed was aware of these guidelines, understood the intention was to promote health, and was the most optimistic of all participants about the way school food tastes.

In contrast, teachers and parents described children as “apathetic” (teacher) about unappealing school lunch foods; the foods are eaten only because they are available when children are hungry. Unappealing, bland food was considered a disservice to children who then do not have a good experience, do not eat, and therefore do not get the nutrition needed to “have that energy to function” (administrator). According to one teacher, “If anything, my fear is if we approach healthy in the wrong way, then they’re not gonna eat it at home either, because they’re gonna say, “oh man, I had that at school once, and it was gross” (teacher).

### Beliefs about ways to increase fruit and vegetable consumption in school meals

**School-level cafeteria strategies to increase fruit and vegetable consumption.** Participants identified numerous cafeteria-based strategies to increase consumption of fruits and vegetables, including making food more visually appealing, tasty, and easier to eat (altering recipes and presentation); enhancements to the cafeteria environment including sampling menu items, explicit staff and teacher verbal encouragement and promotion of fruits and vegetables; and adult modeling of fruit and vegetable consumption.

Teachers spoke about slicing and packaging fruits as a way to get children to eat more. Participants noted that doing so would allow teachers additional time to eat. Additionally, some teachers and administrators said this time could be used to talk to students about the food choices they make, including discouraging unhealthy purchases and encouraging fruit and vegetable consumption.

Suggested changes to the cafeteria environment included promotional efforts. Participants believed making foods fun would make students excited about eating fruits and vegetables. One teacher suggested taking pictures of fruit and vegetable dishes and posting them in the cafeteria to make students look forward to them. Many participants also suggested a greater variety of choices in school lunch, instead of 2 fruits and 2 vegetables. One teacher noted that “it’s a difficult balancing act” to offer many choices and stay within the budget.

**School meal guidelines present perceived barriers to increased fruit and vegetable consumption.** Although implementation of most cafeteria-based strategies proposed by participants would increase school personnel responsibilities, suggested policy solutions would not require additional individual effort.

Participants discussed the portion sizes for younger children and described how a big portion of one food can be intimidating for a small child. In contrast, participants expressed concern that standardized portion sizes meant older children did not get enough food. “[S]ome of my children, in fifth grade — they’re growin’, they’re hitting puberty and they need more food. They’re still gettin’ the same serving size, as a kindergarten child, who’s not eatin’ all that food, but then my fifth graders are eatin’ it all and need more” (teacher).

Standardized portion sizes for every student were of particular concern to teachers because they experienced the consequences of this policy. A fifth grade teacher noted that students said they were hungry because of the limited portion sizes and that younger students were throwing food away because they were served more than they could eat. Mandated portion sizes and restrictions on preparation methods were highlighted as reasons why children do not eat more fruits and vegetables during school lunch.

Many participants believed cafeteria staff and schools are doing the best they can with limited resources and strict guidelines for school food. In general, participants endorsed changes that would make fruit and vegetable consumption appealing and tailor portion size to the child’s age.

## Discussion

Rural adults perceive current school food as bland and aesthetically unappealing and identified several possible ways to increase children’s consumption of fruits and vegetables in school meals. Most notably, these included improving the appearance and taste of menu items, changing existing policies about portion size, and enhancing visual and verbal cues in the environment. To date, there is a paucity of literature on rural stakeholder perspectives on school food. This study fills this gap and provides rural adult perspectives on school food post-HHFKA and insights into ways schools can encourage and reinforce healthy dietary behaviors, all of which are required for building healthy children (23). A coupling of the theoretical literature and the findings from this study suggest numerous promising actions that could be taken at the individual and community level to improve children’s attitudes toward and consumption of fruits and vegetables.

To increase fruit and vegetable consumption, possible individual-level strategies include offering samples of menu items, adults verbally encouraging students to try foods, and increasing students’ knowledge of the benefits of eating fruits and vegetables. Samples and spoken encouragement to try fruits and vegetables from cafeteria staff and teachers are simple, practical strategies that can positively affect a child’s attitude. This study’s results parallel findings from studies of low-income, urban schools where teachers had a significant influence on students’ attitude toward fruits and vegetables through individual-level strategies (24). Successful interventions used classroom curricula to teach students about health-promoting behaviors (12), token reinforcement to increase fruit and vegetable consumption (25), and teacher modeling of daily fruit consumption (26).

School-level strategies include cafeteria promotion of fruits and vegetables, increasing fruit and vegetable choices, making fruits and vegetables easier to eat, and preparing fruits and vegetables to be appealing and taste good. Feasible changes based on these suggestions include placing point-of-sale promotional signs above fruits and vegetables to make them more exciting, preparing tasty and visually appealing foods through recipe adaptations (which may be complicated given current guidelines but could be facilitated by shared recipe ideas and development), and making fruits and vegetables easier to eat through alternative serving methods, such as slicing. Previous interventions made some environmental changes, including increasing availability of healthy food, lowering prices, and point-of-purchase promotional strategies (27), as well as providing technical assistance to school food service personnel (28).

Participants discussed a federal policy change (regulating portion sizes of elementary school lunches) as a barrier to increasing fruit and vegetable consumption. Participants acknowledged that changing portion sizes could affect students of all ages and students’ views on fruits and vegetables in school lunch.

Understanding key informants’ perspectives on school food and strategies to increase fruit and vegetable consumption is a fundamental step in building effective interventions for elementary school children.

Although this study produced themes regarding key informants’ opinions about school food, these findings are from a convenience sample and may not generalize to all populations. Additionally, although telephone interviews were convenient for participants and allowed for greater anonymity, they did not allow interviewers to observe participants’ nonverbal communication. Observing the participants’ nonverbal communication might have enhanced the interview dynamic and led to richer data.

Because school food plays an important role in providing nutrition for students, efforts must be made to increase the appeal and taste of fruits and vegetables offered in school lunch. Although not explicitly stated by participants, creating flavorful and visually appealing dishes that abide by HHFKA is a challenge because the cafeteria staff has historically seasoned dishes with butter and salt. Future policy interventions may consider altering portion sizes depending on the age of the student. Finding a balance between HHFKA, making food appealing to look at, and positive messaging in cafeterias may begin to increase children’s consumption of fruits and vegetables.
